# Yield-determining factors in high-solids enzymatic hydrolysis of lignocellulose

**DOI:** 10.1186/1754-6834-2-11

**Published:** 2009-06-08

**Authors:** Jan B Kristensen, Claus Felby, Henning Jørgensen

**Affiliations:** 1 Forest and Landscape Denmark, University of Copenhagen, Rolighedsvej 23, DK-1958 Frederiksberg, Denmark; 2 Novozymes A/S, Krogshøjvej 36, DK-2880 Bagsværd, Denmark

## Abstract

**Background:**

Working at high solids (substrate) concentrations is advantageous in enzymatic conversion of lignocellulosic biomass as it increases product concentrations and plant productivity while lowering energy and water input. However, for a number of lignocellulosic substrates it has been shown that at increasing substrate concentration, the corresponding yield decreases in a fashion which can not be explained by current models and knowledge of enzyme-substrate interactions. This decrease in yield is undesirable as it offsets the advantages of working at high solids levels. The cause of the 'solids effect' has so far remained unknown.

**Results:**

The decreasing conversion at increasing solids concentrations was found to be a generic or intrinsic effect, describing a linear correlation from 5 to 30% initial total solids content (w/w). Insufficient mixing has previously been shown not to be involved in the effect. Hydrolysis experiments with filter paper showed that neither lignin content nor hemicellulose-derived inhibitors appear to be responsible for the decrease in yields. Product inhibition by glucose and in particular cellobiose (and ethanol in simultaneous saccharification and fermentation) at the increased concentrations at high solids loading plays a role but could not completely account for the decreasing conversion. Adsorption of cellulases was found to decrease at increasing solids concentrations. There was a strong correlation between the decreasing adsorption and conversion, indicating that the inhibition of cellulase adsorption to cellulose is causing the decrease in yield.

**Conclusion:**

Inhibition of enzyme adsorption by hydrolysis products appear to be the main cause of the decreasing yields at increasing substrate concentrations in the enzymatic decomposition of cellulosic biomass. In order to facilitate high conversions at high solids concentrations, understanding of the mechanisms involved in high-solids product inhibition and adsorption inhibition must be improved.

## Background

Climate changes and shortage of fossil fuels have sparked a growing demand for liquid biofuels which in turn has increased the amount of research into the production of lignocellulose-derived bioethanol [1,2]. However, being an insoluble and highly heterogeneous substrate, lignocellulosic materials pose several challenges in conversion to fermentable sugars. In addition to understanding complex enzyme system kinetics, these biomass-related challenges include recalcitrance to hydrolysis [3] and mixing difficulties [4]. Water content in the hydrolysis slurry is directly correlated to rheology, that is, viscosity and shear rate during mixing [5], important for the interaction between lignocellulose and cell wall-degrading enzymes. Thus, water is not only critical in hydrolysis being a substrate and a prerequisite for enzyme function, but is also crucial for enzyme transport mechanisms throughout hydrolysis as well as mass transfer of intermediates and end-products [6]. Maintaining high substrate concentrations throughout the conversion process from biomass to ethanol is important for the energy balance and economic viability of bioethanol production.

High-solids enzymatic hydrolysis can be defined as taking place at solids levels where initially there are no significant amounts of free liquid water present [7]. By increasing the solids loading, the resulting sugar concentration and consequently ethanol concentration increase, having significant effects on processing costs, in particular distillation [8-10]. Furthermore, lower water content allows for a larger system capacity, less energy for heating and cooling of the slurry and less waste water [4]. Model-based estimations have shown significant reductions of operating costs of simultaneous saccharification and fermentation (SSF) of pretreated softwood when the initial solids concentration was increased [8]. Unfortunately, there are also disadvantages to increasing the substrate concentration. Concentrations of end products and inhibitors will increase, causing enzymes and fermenting organisms to not function optimally. Also, high-solids loadings can cause insufficient mixing, or mixing can be too energy-consuming in conventional stirred-tank reactors as the viscosity of slurries increases abruptly at increasing solids loadings, in particular over 20% solids [11,12].

*In situ *native cellulase systems have been reported to function at solids levels as high as 76% (all concentrations are given as total solids on a *w/w *basis) [13], indicating that enzymatic hydrolysis may be limited by the laboratory or industrial process set-up. Twelve to fifteen per cent total solids is often considered the upper limit at which pretreated biomass can be mixed and hydrolysed in conventional stirred-tank reactors [7,14,15]. However, at the laboratory scale, enzymatic hydrolysis at up to 32% total solids has been reported [12,16]. A number of studies have utilised fed-batch operations in order to increase the final solids loading [7,11,17,18]. We have previously described a gravimetric mixing reactor design that allows batch enzymatic liquefaction and hydrolysis of pretreated wheat straw at up to 40% solids concentration [4]. This is a significant increase from what has previously been possible, and thus significantly increases the techno-economic potential of the whole process. The gravimetric mixing principle has been up-scaled and used in a pilot plant for several years [19,20].

**Figure 1:**
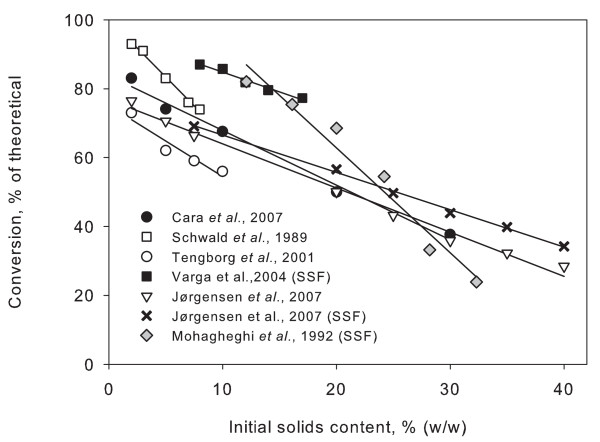
**Decreasing conversion of biomass**. Results collected from several publications indicate that decreasing conversion at increasing solids content is a general effect. Results are for different kinds of biomass and for both enzymatic hydrolysis and simultaneous saccharification and fermentation (SSF). Added trend lines show that for each experiment there is a linear relationships between initial solids content and yield. Data taken from [24] (enzymatic hydrolysis), [16] (enzymatic hydrolysis), [23] (enzymatic hydrolysis), [17] (SSF), [4] (enzymatic hydrolysis and SSF) and [12] (SSF).

During the work with high solids concentrations we found that the enzymatic conversion (percent of theoretical) linearly decreased with increasing solids concentration (constant enzyme to substrate ratio) [4]. This decrease partly offsets the advantages of running at high solids concentrations. As seen in Figure 1, the effect has been observed in both enzymatic hydrolysis and SSF by several groups working with various kinds of biomass [12,16-18,21-24]. Although several of these studies were conducted at less than 10% initial solids content, the phenomenon appears to be an intrinsic or generic effect of enzymatic hydrolysis at increasing solids levels. In this paper, the decrease in yield at high solids concentrations is referred to as the solids effect.

Some groups have suggested that the mechanism behind the decreasing conversion is product inhibition [12,16,25] or inhibition by other compounds such as sugar-derived inhibitors (furfural and hydroxymethylfurfural (HMF)) [26] and lignin [27]. Others have suggested it may be explained by mass transfer limitations or other effects related to the increased content of insoluble solids, such as non-productive adsorption of enzymes [14,28]. However, the specific mechanism(s) responsible for the decreasing hydrolytic efficiency are still uncertain [4,29].

It should be noted that inhibition primarily affects the hydrolysis rate and not the maximum conversion or yield, given sufficient time. With limited reaction times and not fully converted, the conversion will correspond to the inhibition, that is, the conversion being a measure of the 'accumulated' inhibition. Working with initial reaction velocities in high-solids hydrolysis involves great difficulties due to the non-liquid properties of the substrate. For that reason, degree of conversion has been used to estimate the increased inhibition that appears to take place at elevated solids contents.

In this paper the possible mechanisms behind the solids effect have been divided into the following four categories: Compositional and substrate effects; product inhibition; water concentration; and cellulase adsorption. These four topics will be introduced below.

### Compositional and substrate effects

The heterogeneity and structure of lignocellulosic biomass means that high viscosity prevents efficient mixing at high solids concentrations when performed in conventional stirred-tank reactors [14,28,30]. The viscosity of lignocellulosic slurries increases sharply over a certain threshold (typically around 20% solids) but, despite the extreme difference in viscosity between, for example, 5% and 40% solids loading, the conversion of lignocellulosics as a function of solids appears to be linear (Figure 1). Although mixing of substrate and enzymes is crucial for an efficient liquefaction, our previous findings showed that it does not appear that lack of mixing is the cause of the decreasing conversion, at least not at the solids levels documented [4]. This is in accordance with the recent findings of Hodge and co-workers who concluded that possible mass transfer limitations caused by insoluble solids were not apparent at below 20% insoluble solids content [25]. At very high solids levels (above 20 to 30% dry matter), a mass transfer limitation may be involved in the lower yield, but the linearity of the solids effect over a large range of conditions with a number of substrates (wheat and barley straw [4,12,14], corn stover [17], softwood [22,24], hardwood [16,23] and an industrial ethanol fermentation residue (vinasse) [28]) indicates that a single factor may be responsible for the effect (all the way from, for example, 5% to 40% dry matter).

In order to be able to establish that the solids effect is not caused by lignin adsorption or lignin-derived inhibitors (phenolics), experiments for this paper were carried out with filter paper. Filter paper has the advantage of containing no lignin yet still retains the secondary cell wall structure, as opposed to Sigmacell and Avicel, for example.

### Product inhibition

End-product inhibition plays an important role in enzymatic hydrolysis as glucose, cellobiose and ethanol have demonstrated their ability to significantly inhibit endoglucanases, cellobiohydrolases and *β*-glucosidase [31,32]. However, working with an insoluble substrate and kinetics that do not follow the Michaelis-Menten model, the exact type of inhibition is difficult to determine [33]. The decrease in hydrolysis rate over time has been attributed to inhibition by the accumulated end products [34]. Others conclude that when hydrolysing natural, lignocellulosic substrates, cellulases are more resistant to product inhibition than with amorphous reference materials and that the early stage decrease in hydrolysis rate is not caused by product inhibition [35,36]. In high-solids enzymatic hydrolysis of pretreated corn stover, Hodge and co-workers recently found that increased sugar concentrations were the primary cause of performance inhibition [25]. Based on the above, we have investigated the inhibitory effect of increased sugar concentration in connection with high-solids enzymatic hydrolysis.

### Water concentration

Working with a system with low water content may directly affect enzyme performance. Not only is water a substrate for the hydrolysis but it is also the solvent that allows the function of enzymes, contact between enzymes and substrate and transport of products [37]. We have previously investigated the role of water in enzymatic hydrolysis [6]. In this study, we wanted to investigate if the solids effect was related to a lower concentration of water in relation to solids. As mentioned, hydrolysis is possible at very high solids concentrations but the rate of reaction may be impaired under such conditions [13].

We have investigated the role of water concentration by replacing various amounts of the water in enzymatic hydrolysis with oleyl alcohol, an inert oil that does not directly affect the function of the enzymes [38,39].

### Cellulase adsorption

Cellulose accessibility and degree of adsorption of cellulases are well known as controlling factors for conversion rates and yields [40,41]. It has long been known that certain hydrolysis products are able to inhibit cellulase adsorption [42]. It has, however, recently been shown that glucose and especially cellobiose strongly inhibit cellulase adsorption in a linear fashion [43]. This adsorption inhibition can be seen as a sub-class of product inhibition where the catalytic site may not necessarily be involved. In order to investigate whether adsorption (or lack thereof) could possibly be involved in the observed solids effect, the adsorption of enzyme was measured in hydrolysis of filter paper at different solids contents.

## Results and discussion

### Compositional and substrate effects

**Figure 2:**
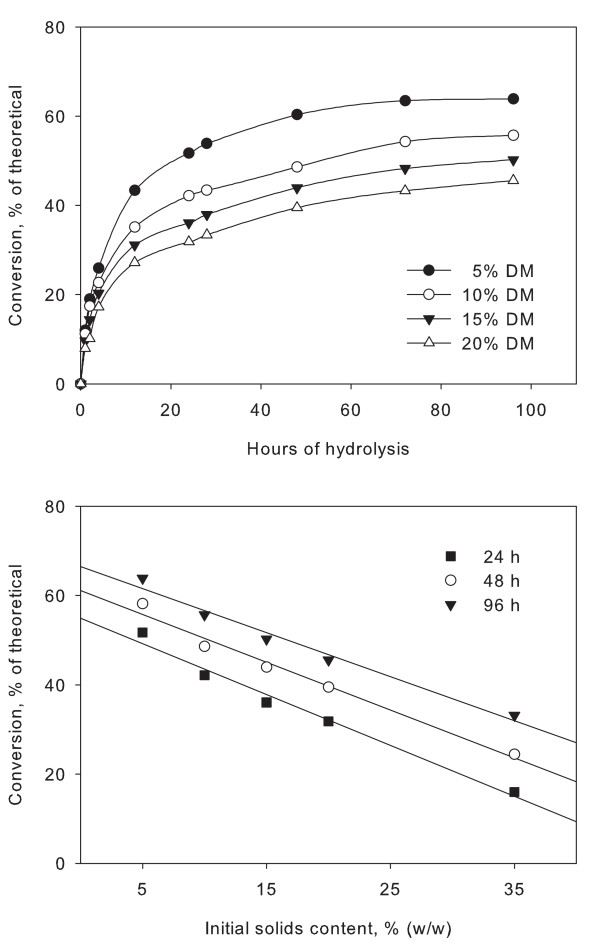
**Hydrolysis of filter paper**. Hydrolysis of filter paper at large laboratory scale with 5, 10, 15, 20 and 35% initial solids content (w/w) and an enzyme dosage of 10 FPU per gram dry matter (DM). A: Hydrolysis profiles for 5, 10, 15 and 20% DM as a function of time. B: Cellulose conversion as a function of initial solids concentration.

Filter paper was used as a model substrate. As seen in Figure 2A, filter paper hydrolysis displayed the characteristic profiles, with a very high initial rate of conversion that decreases considerably after only 6 to 8 h. When the conversion was displayed as a function of initial solids content, the characteristic downward curve was observed (Figure 2B). Again, the relationship is linear with a decrease from 56.5% conversion at 5% initial solids content to 22.8% conversion at 25% initial solids content, both after 24 h of hydrolysis at large laboratory scale (see explanation of 'small' and 'large' laboratory scale in the Methods section). The 5% solids conversions shown in Figure 2B are slightly higher than the linear curve. This observation is not in accordance with previous results of hydrolysis at different scales and is possibly a measurement artefact [4,44]. Numerous other experiments have been performed with filter paper (not shown). As above, they all exhibited the same solids effect as observed with a range of lignocellulosic substrates with varying lignin content. Based on this it is unlikely that lignin or other phenolics are responsible for the solids effect.

The filter paper used in the experiments for the present paper contained approximately 15% hemicellulose in the form of 14% mannan and 1% arabinan. However, experiments with hydrolysis of Whatman filter paper (98% cellulose) (not shown) and hydrolysis of α-cellulose also displayed the same trend at increasing solids loadings [21]. As regarding lignin, the fact that a hemicellulose-free substrate exhibits the same trend at increasing solids contents indicates that hemicellulose-derived sugars/inhibitors are not the cause of the solids effect either.

### Product inhibition

**Figure 3:**
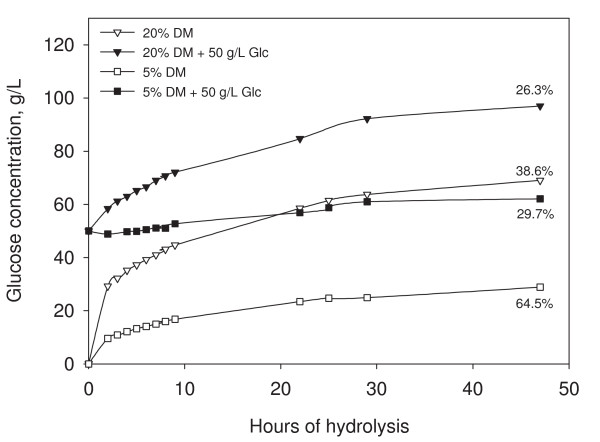
**Hydrolysis of filter paper**. Hydrolysis of filter paper at large laboratory scale with 5% (punctuated line) and 20% (solid line) initial solids content (w/w) and an enzyme dosage of 10 FPU per gram dry matter (DM). Before addition of enzyme, 50 g/l glucose was added to the substrate (open symbols). The references with no sugar addition are depicted with solid symbols. The final degree of conversion is indicated for each experiment.

To investigate the role of product inhibition in high-solids enzymatic hydrolysis, various amounts of sugar were added to a hydrolysis of filter paper. An example of such an experiment (at large laboratory scale) is seen in Figure 3. With 50 g/l glucose added, the rate of hydrolysis during the first few hours was significantly reduced compared with the reference, in particular for the 5% solids hydrolysis where the initial phase of fast conversion was completely absent. As there is a constant enzyme dosage per gram of solids in the experiments, the ratio between glucose and enzyme is much higher at 5% than 20% solids (for the hydrolyses with 50 g/l glucose added) and the stronger inhibition is thus not surprising. Although 4 h makes up a small part of the whole hydrolysis time, the fast rate of hydrolysis in the first phase is responsible for conversion of a major part of the substrate. Interestingly, after approximately 4 h, the rate of hydrolysis at 20% is nearly identical despite the difference in glucose level. This indicates that one of two things is happening. Either there are other and stronger factors inhibiting the hydrolysis after the first phase, thereby 'masking' the product inhibition, or there is a certain glucose level threshold, above which the enzymes are inhibited to a similar extent and thus resulting in a similar hydrolysis rate.

It is worth noting that it is not only the concentration of the inhibitor that is important but the inhibitor-to-enzyme ratio should also be considered. Depending on the difference in concentrations of substrate and enzymes and thus their collision rate, the inhibitor-to-enzyme level can determine the degree of inhibition. Xiao and co-workers showed that in hydrolysis of a cellobiose solution, addition of 20, 50 and 100 g/l of glucose to 2, 5 and 10% cellobiose (w/v) resulted in β-glucosidase inhibition of 53, 51 and 48%, respectively. The almost identical degree of inhibition at different sugar concentrations shows that the inhibitor-to-enzyme ratio is essential in product inhibition [32]. Based on this, it does not appear likely that inhibition of β-glucosidase is the main cause of the solids effect. However, indirectly the cellobiohydrolases are even stronger inhibited by glucose. The high glucose concentration inhibits β-glucosidase, which in turn leads to an accumulation of cellobiose, which acts as a particularly strong inhibitor of cellobiohydrolases [33].

Surprisingly, cellobiose concentrations in our experiments have generally been low. Normally, even at high solids concentrations and 80% conversion, less than 10% of the converted material is found as cellobiose (data not shown). For comparison, during experiments with lower proportions of β-glucosidase, inhibition caused cellobiose proportions of over 35% of the converted material while still retaining a certain degree of hydrolysis (data not shown).

SSF is normally used to offset the well-known effects of glucose and cellobiose inhibition but interestingly the solids effect has also been observed under those conditions [12,17,19]. Ethanol is also known to act as an inhibitor of cellulases (although less severe an inhibitor than cellobiose) [31,45], indicating that other factors may influence the conversion under these conditions.

**Figure 4:**
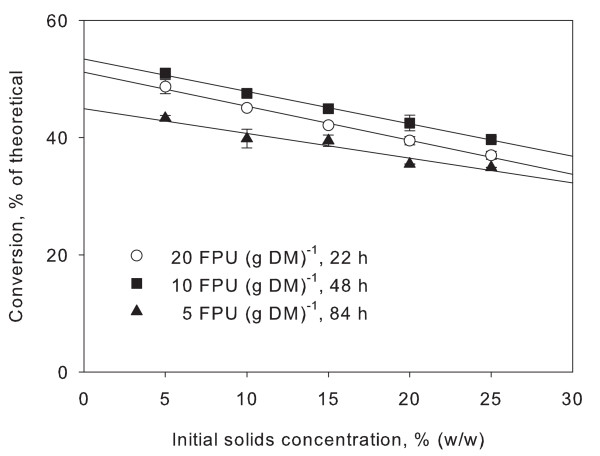
**Enzyme loadings**. Filter paper was hydrolysed at small laboratory scale to approximately the same extent by using three different enzyme loadings and lengths of hydrolysis time: 20 FPU per gram dry matter (DM), for 22 h, 10 FPU per gram dry matter, for 48 h and 5 FPU per gram dry matter for 84 h. Points are averages of three observations. No significant difference in slope of the curves at the different enzyme loadings was observed.

To test if product inhibition was the sole cause of the solids effect a new experiment was carried out. Filter paper was hydrolysed using three different combinations of enzyme loading and time: 20 FPU (g DM)^-1 ^for 22 h, 10 FPU (g DM)^-1 ^for 48 h and 5 FPU (g DM)^-1 ^for 84 h. This was done in order to reach approximately the same degree of conversion (45%) despite using different enzyme loadings. This means that the same amount of sugar was released in all three experiments. Theoretically this amount of sugar should cause a larger degree of inhibition on a small amount of enzyme (low enzyme dosage but longer hydrolysis time) *versus *a larger amount of enzyme (high enzyme dosage but short hydrolysis time) as per the inhibitor-to-enzyme ratio previously discussed. As seen in Figure 4, the slopes of the three curves are nearly identical. If product inhibition alone was the cause, one would expect the hydrolysis with the lowest enzyme-to-substrate ratio (that is, lowest enzyme dosage) to display the strongest degree of inhibition and thus a steeper curve. In other words, it is not possible to bypass the solids effect by using higher enzyme dosages, at least not within the normal range of dosages. This is an important consideration when trying to alleviate the solids effect.

In conclusion, product inhibition at increased solids concentrations was found to be a significant and potentially determining factor for the solids effect. However, the linearity over a large range of solids contents of our experiments does not fit with the current models for product inhibition.

### Water concentration

Oleyl alcohol has previously been shown to exhibit partitioning behaviour towards water and sugars [39] and our experiments showed no detrimental effects on enzyme performance (data not shown). Therefore, it was possible to use oleyl alcohol to replace water in order to investigate the water-to-enzyme/solids ratio while keeping the viscosity similar. The reasoning behind these experiments is that by substituting part of the water, it is possible to run a hydrolysis with an altered water-to-enzyme ratio but with a more-or-less constant viscosity of the slurry. If a lack of water is causing the solids effect, then the hydrolysis conversion where a certain amount of the water has been replaced should be lower, presumably at the level of the corresponding solids level (taking only the aqueous phase in consideration).

**Figure 5:**
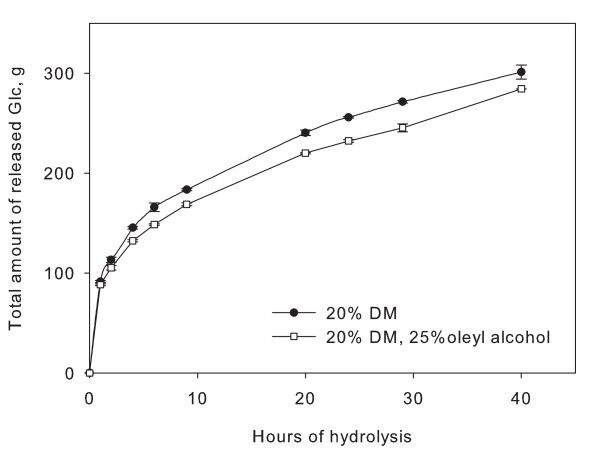
**Replacement of water**. Hydrolysis of filter paper at large laboratory scale with 20% initial solids content (w/w) and an enzyme dosage of 10 FPU per gram dry matter (DM) where 25% of the water was replaced with the inert oil, oleyl alcohol. This corresponds to an increase of biomass to water ratio of 25%. The yield was found to be less than the reference, but not as low as a 25% increase in solids normally results in. Points are averages of two experiments.

In Figure 5, a quarter of the water (buffer) in an enzymatic hydrolysis of 20% solids filter paper has been substituted. At this level of substitution, the actual solids concentration in relation to water has therefore been increased from 20 to 25%. After 40 h of hydrolysis, 5.6% less glucose compared with the reference (without oleyl alcohol addition) was released. However, the increase from 20 to 25% solids usually leads to a decrease in conversion of over 12%. Thus, the decrease in conversion did not correspond directly to the lowered water content.

However, the sugar concentration is not the only parameter that has been changed. Oleyl alcohol may act as a mixing agent, fully or partially replacing the effect of water in assisting mass transfer, even if neither enzymes nor sugars can be solubilised in oleyl alcohol. As previously discussed, the interconnection of factors affecting the yield is characteristic of lignocellulose hydrolysis, complicating the identification of limiting factors.

There is no doubt that water plays a number of important roles in enzymatic hydrolysis, and that these roles become even more crucial in systems with no free water. As cellulases can only break down cellulose when adsorbed onto the material, efficient mass transfer of enzymes is likely to increase conversion. Also, diffusion of released sugars away from the catalytic sites will theoretically prevent local product inhibition. Mechanical stirring may also directly change the size distribution of larger particles. Unfortunately, our understanding of these mechanistic interactions is limited and also depends on the cell wall structure of the substrate. It is likely that such factors affect the degree of conversion at very high solids loadings, essentially causing a drop-off in yield over a certain solids loading. However, as already discussed, the observed solids effect is also seen at loadings as low as 2 to 5% solids and thus mass transfer at neither the macroscopic nor the molecular level appears be responsible for the solids effect.

Related to the diffusion of enzymes is the phenomenon of substrate inhibition, which has previously been described in connection with hydrolysis of cellulose [46]. At increased substrate concentrations, with a fixed enzyme loading, the lateral (two-dimensional) diffusion of bound enzymes is believed to be restricted, thus inhibiting the synergy between exo and endo-enzymes [47]. However, this form of synergistic inhibition relates to a fixed enzyme load where the amount of substrate is increased, that is, a decreasing enzyme-substrate ratio as opposed to a constant ratio as used in our and other's experiments. Therefore, this phenomenon is not likely to be involved in the solids effect. Traditionally, substrate inhibition is explained as a situation where two molecules of substrate bind to the enzyme simultaneously, thereby blocking activity. However, this mechanism is not likely to be applicable to the hydrolysis of cellulose due to its insoluble nature [48].

In conclusion, water itself as a substrate or diffusing agent in enzymatic hydrolysis does not appear to be the limiting factor responsible for the solids effect, nor is substrate inhibition involved.

### Adsorption

**Figure 6:**
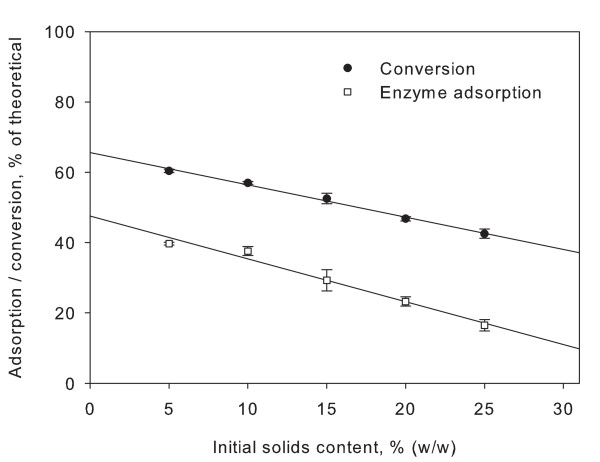
**Enzyme adsorption**. Upper graph shows the decreasing conversion in enzymatic conversion of filter paper at increasing solids loading (20 FPU per gram dry matter (DM), 24 h hydrolysis at small laboratory scale). Points are averages of three observations. The lower graph shows the adsorption of enzyme on the solid fraction based on total nitrogen content, also as a function of initial solids content. Values are averages of three observations and have been corrected for varying amounts of remaining solids.

Based on previous reports on inhibition of enzyme adsorption, it was investigated if the increased sugar concentration at high solids concentration could cause the solids effect in this manner [42,43]. As seen in Figure 6, there is a linear correlation between initial solids content and amount of adsorbed enzyme (percentage of nitrogen adsorbed on solids of total nitrogen added). After 24 h of hydrolysis of 5% solids filter paper, approximately 40% of the added enzyme was adsorbed onto the remaining solids. The adsorption decreases with increasing solids content, and at 30% solids content only approximately 17% of the added enzyme is adsorbed, despite significantly more solids remaining than at lower solids contents. Even more interestingly, linear regression of sample pairs reveals a statistically significant correlation between the decrease in conversion and the decrease in enzyme adsorption. In other words, it appears that the increasing concentrations of glucose and cellobiose in high-solids hydrolysis result in inhibition of adsorption of the enzymes. As adsorption is a requirement for hydrolysis of the insoluble substrate, this in return results in lower conversion at increasing solids concentrations.

Based on an experiment with a fixed cellobiose concentration, Kumar and Wyman argue that binding inhibition can be reversed using high substrate concentrations [43]. However, working with a fixed inhibitor concentration over a range of solids concentrations does not reflect the actual conditions since high solids loadings will invariably lead to higher product concentrations. At any degree of conversion, the ratio between substrate and inhibitor (product) in hydrolysis will be constant no matter the initial solids concentration. Xiao and co-workers also observed reduced impact of products on inhibition at higher solids loadings, but again it was measured against a constant inhibitor concentration [32]. Based on our experiments we do not believe that increased solids concentrations can reverse binding inhibition, rather the opposite.

It can be argued that the phenomenon described above is a variant of product inhibition. In both competitive and non-competitive inhibition the catalytic site is affected, which is not necessarily the case with inhibition of adsorption. Although β-glucosidase does not bind to the substrate and thus is not affected in this way, the binding inhibition of endoglucanases and cellobiohydrolases can possibly explain the low cellobiose levels under conditions where hydrolysis is inhibited.

It is not known to what extent inhibition of adsorption is responsible for the solids effect, or if it can be partially avoided through SSF. It has previously been shown that adsorption inhibition could not explain the decrease in cellulase activity [49]. In an attempt to learn more about the nature of the inhibition, we used the data of the experiment in Figure 2 to investigate the relationship between the rate of reaction and glucose concentration. We found no direct relationship (data not shown), possibly due to the fact that different proportions of the substrate remained, that is, when 50% of the substrate has been converted, the remainder is more difficult to hydrolyse.

It is likely that the cellulose binding domains (CBD) of the cellulases are affected by glucose and cellobiose. Binding of cellulases and clarification of the role of CBDs is an important topic in cellulosic biomass conversion, and has been the topic of numerous studies. Being able to alter the CBD to make it less susceptible to a high concentration of products may contribute to making high yields at high solids concentrations a reality.

## Conclusion

The extent of enzymatic conversion of cellulosic biomass was investigated at varying solids concentrations. The conversion decreased at increasing solids concentration in a linear fashion, an effect that appears to be a generic or intrinsic feature of lignocellulose conversion. This decrease partially offsets the significant advantages of working at high solids concentrations. The solids effect did not appear to be caused by lignin content or hemicellulose-derived inhibitors. Lack of mixing of the insoluble substrate did not appear to be causing the effect either.

The increased concentration of glucose and cellobiose at high solids concentration are likely to cause product inhibition even when the enzyme-to-inhibitor ratio is constant. However, the solids effect has also been observed in SSF where much less sugar is present.

It was found that at increasing solids concentrations, the proportion of adsorbed cellulase decreased. There was a statistically significant correlation between this adsorption inhibition and the decreasing yields at increasing substrate concentrations. Thus, the solids effect may well be explained by inhibition of the binding of the cellulases. The exact extent and mechanism of the adsorption inhibition is still unknown. It is possible that improvement of cellulase CBDs may lead to enzymes that are more resistant to high sugar concentrations and thus higher conversions at high solids concentrations, significantly improving the viability of lignocellulosic biomass conversion.

## Methods

### Compositional analysis

The composition of filter paper (AGF 725, 140 g/m^2 ^from Frisenette ApS, Knebel, Denmark) was analysed using two-step acid hydrolysis according to the procedure published by NREL [50]. Dry matter content was determined using a Sartorius MA 30 moisture analyser at 105°C. The released sugars were quantified by high performance liquid chromatography (HPLC) as described below. The filter paper was found to consist of 80.6% glucan, 0.42% Klason lignin, 14.4% mannan, 1.0% arabinan, and 0.24% ash.

### Enzymatic hydrolysis

The hydrolyses were performed using an enzyme mixture of Celluclast 1.5 L and Novozym 188 (weight ratio 5:1, both from Novozymes A/S, Bagsværd, Denmark) with a filter paper activity of 75 FPU per gram of dry matter (DM), as measured by the filter paper assay [51]. Enzyme loadings of 5 to 20 FPU per gram of DM and a hydrolysis times from 24 to 84 h were used. Hydrolysis temperature was 50 ± 1°C. Initial total solids content ranged from 5 to 35% (w/w) and pH was kept constant by adding sodium citrate buffer (pH 4.80, 50 mM final concentration).

Hydrolysis experiments were performed at one of two scales. The 'large' scale hydrolyses were done in a horizontal, five-chambered liquefaction reactor where each chamber is 20 cm wide and 60 cm in diameter as described in [4]. In this reactor, a total reaction mass (solids and liquids) of 5 kg was used. The rotational speed was approximately 6 rpm.

The 'small' scale hydrolysis was performed in 100 ml plastic bottles (total reaction mass 50 g), also at 5 to 25% solids content (w/w); buffer concentration and enzyme loadings as described above. The bottles were placed in a heated, horizontally placed drum, rotating at 60 rpm. The 80 cm diameter drum was equipped with two inside paddles that lifted and dropped the plastic bottles during rotation, mimicking the gravimetric mixing described in [4,20]. All small-scale experiments were performed in either duplicate or triplicate.

Samples for HPLC sugar analysis were boiled for 10 min to terminate the reaction. Whole slurry was sampled after vigorous shaking to ensure a representable mixture of solids and liquid. Samples were then diluted five to tenfold with eluent before insoluble material was removed by centrifugation at 4,200 × g for 10 min. The dilution factor was determined by measuring the weight of the sample before and after dilution. When working at high insoluble solids concentrations there is an increasing difference between the concentration in the liquid phase and the overall concentration of a component [7]. The dilution step minimises the measurement error introduced by the content of insoluble material, which would otherwise result in an overestimation when calculating the conversion, as discussed in [44].

### Sugar analysis

The content of monosaccharides in the hydrolysed samples (D-glucose, D-xylose, L-arabinose and D-cellobiose) was quantified on a Dionex Summit HPLC system equipped with a Shimadzu RI-detector. The separation was performed in a Phenomenex Rezex RHM column at 80°C with 5 mM H_2_SO_4 _as eluent at a flow rate of 0.6 ml min^-1^. Samples were filtered through a 0.45 μm filter and diluted with eluent before analysis on HPLC.

### Inhibition experiments

Before hydrolysis, various amounts of D-glucose (Sigma-Aldrich, Brøndby, Denmark) were added to the substrate. Conditions were as described above.

### Water replacement experiments

Hydrolysis was run at 'large' scale, as described above, with 20% solids content (w/w) and an enzyme loading of 10 FPU (g DM)^-1^. Twenty-five per cent (w/w) of the initial aqueous phase was substituted with oleyl alcohol. It was found that neither the enzyme nor the released sugars was present in the oleyl alcohol. Sugar concentration was measured in the aqueous phase only.

### Adsorption experiments

For cellulase adsorption studies, samples were kept on ice after hydrolysis instead of boiling, in order to prevent any desorption of enzyme from the solids. Rather than estimating the adsorption indirectly with a colorimetric method, total nitrogen content of the biomass was determined on an elemental analyser coupled to an isotope ratio mass spectrometer (ANCA SL & 20–20, Europa Scientific, Crewe, UK). This method of measuring enzyme adsorption has recently been described by Kumar and Wyman [43]. As the cellulase mixture of Celluclast 1.5 L and Novozym 188 contains a proportion of non-binding enzymes, enzyme adsorption will never reach 100% of the added amount. To be able to subtract the nitrogen content of the liquid of the spun-down samples, the nitrogen content of the aqueous phase was measured with the Kjeldahl method.

## Competing interests

The authors declare that they have no competing interests.

## Authors' contributions

JBK carried out the majority of the experiments and drafted the manuscript. CF participated in the inhibition work as well as design and coordination of the study. HJ participated in the general hydrolysis work as well as the experimental design and helped draft the manuscript. All authors suggested modifications to the draft, commented on several preliminary versions of the text and approved the final manuscript.
